# Food of Infants

**Published:** 1885-11

**Authors:** Fred. Hooper Hayes

**Affiliations:** Dover, N. H.


ARTICLE V.

### FOOD OF INFANTS.

BY FRED. HOOPER HAYES, D. D. S., DOVER, N. H.

The milk of the mother is the natural and only proper food for an infant. Therefore, the child should be entirely confined to it till the process of dentition has made some progress. I do not profess to be an authority, but I do feel that it is one of great interest to every practitioner of dentistry. We should be as well qualified to give advice and recommend a judicious course to be followed in this, as in any other branch of our profession. At birth the child has the germs of all the teeth. It is therefore very essential that special attention should be given to the period of gestation, and that the mother should feed on the most nourishing and lime-producing food.* Much of the food that is daily consumed has lost nearly all of the phosphates so necessary for the development of the teeth and bones.

Very little attention is paid by the profession to the food of infants, and to infant hygiene. As a general rule, subject to a few exceptions, the mother's milk only will afford, during the first eight, ten or twelve months of existence, adequate nourishment, and is the best adapted to promote a proper growth of an infant's body. Food must vary in different periods of life. The infant needs a fattening diet; and this is supplied in the milk of the mother, which contains more of the butter (fattening portion) than does the milk of any other animal. The infant has much less exercise than the young of other animals, and does not require so much *azotized food*. Mortality diminishes with every day of advancing life. In the first year of life the stomach and intestines, in the second, the bronchi and lungs, are the sources of increase of the death rate.

It has ever been a problem to provide a suitable food for the young, wholly or partially deprived of its natural nourishment—the mother's milk. The nearer the substitute is to this, the better. When the milk furnished by the mother is too small in quantity, though good in quality, the infant should be supplied partly with cow's milk. By diluting it the proportion of caseine can be made to correspond with that in the mother's milk,but the proprotion of albumen becomes less. Caseine is the pure curd of milk, and consists of carbon, hydrogen, nitrogen, oxygen, and a small proportion of sulphur. Albumen is easily digested, but the caseine becomes indigestible, being coagulated by the acids of the stomach. By the addition of water, the sugar, which is neessary for bodily heat, becomes less. Cane sugar will not answer, as, in order to be assimilated, it must first be converted into grape sugar by the fluids of the digestive organs, and these fluids are not secreted in sufficient quanity by infants. It also produces, by fermentation, acids in the stomach which result in irritation. The presence of albuminoids is of the utmost importance in the nutrition of infants, as well as adults. Sugar should not be given as the principal food, but as an addition to less palatable articles of diet. Milk is supplied by nature to be our first food. This fact receives but little consideration by the dental profession. It contains representatives of all the substances which make the animal frame, and it is on this account that it occupies so high a position among articles of food. It contains curd, which has nitrogen, and is equivalent to albumen and fibrine, and represents the blood formers. It has butter and sugar. These represent the heat formers. It has salts—potash, soda, phosphorus, etc.

The spontaneous acidification of the milk is caused by the fermentation of the sugar of milk, under the influence of the caseine, which results in the production of lactic acid. Lactic acid is an important constituent of the animal body, being found in the juice of muscular flesh, and in the gastric juice. Food will be valuable in proportion as it combines, in proper proportion, the articles contained in the four groups, represented by albumen, fat, sugar, and salts.

All animal and vegetable life begins with the cell. No cell is formed without a minute particle of oil. The portion not used in forming cells is either burned to keep us warm, by uniting with oxygen, or is stored in the cellular tissue, adding to the bulk of the person. As the very beginning of life is dependent on fat, it is of importance as an article of diet. When not taken in as food, it is formed out of albumen, in the process of assimilation. Starch and sugar are never used in forming the tissue; but they perform important offices in the changes going on in the human organism. Wheat contains albumen, fat, sugar, and salts in excellent proportion. When not deprived of the bran, it is perhaps the very best supporter of animal life. For this reason it is called "the staff of life."

Scarcely anything in the form of food could be devised that is so little adapted for nourishment during the first months of existence, or more liable to produce derangement of the stomach and the infant's health generally, than the compounds of flour and milk, oatmeal and water, and the like. The powers of the infant's stomach are inadequate to digest properly the articles of food thus forced on it, and consequently results in gastric irritation, griping pains, disturbed sleep, and emaciation. The little sufferers often, too often, are forced to take more of the same food to relieve these troubles, or are given some carminative or cordial, and thus the mischief is constantly increased, till some severe disease is induced. A few vigorous children pass through these distresses produced by improper food without sustaining permanent injury to the health; often, however, the gastric irritation does not easily pass off and other dangerous maladies result. Food improperly digested results in improper fermentation and decomposition. In children it causes sour stomach and vomiting.

There is little danger to the teeth when the child is supplied with its mother's milk. But all the tissues get just the nourishment they need. The different parts of the body require different materials for their formation and sustenance.

As a general rule, it is a duty incumbent on every mother to nourish her own infant. Occasionally the mother is incapacitated by disease, or a defect in the nutritive properties of her milk, and it becomes necessary to transfer her offspring to the care and nourishment of a nurse, or to resort to cow's milk. A course should be followed which is best calculated to produce the best results in the dental structures. When "infant's food" is added to the cow's milk, it should be one containing a sufficient quantity of the phosphates which so powerfully assist in the proper formation of the teeth and bones. The utmost care should be taken to keep the sucking bottle perfectly clean and free from acidity. The danger which attends every attempt to rear an infant by hand, as it is termed, is now very generally recognized by mothers.—*Ohio State Journal.*

